# Health Tract, No. 160

**Published:** 1865

**Authors:** 


### HEALTH TRACT, NO. 160.

### ONE BY ONE.

"One by one the leaves are falling,
One by one the moments fly;
Thus to thoughtless mortals calling,
They may soon be called to die."

As our minister was ascending the pulpit on a beautiful and bright Sunday morning of the mellow autumn, the thought occurred to us: "Will he ever die?" He had been doing the same thing for many, many years; and in all that time did not seem to have become any older; yet we knew there was a fatal canker at the root; the next summer he died! And there was the mother of Isabella Graham. She sat in the same pew with us. Time passed on. Neither did she seem to be getting any older; and when her minister would come down from the pulpit after service she would make her way through the crowd to shake hands with him, as if to say: "I have been fed to-day." One day she was seen to be at unusual pains to greet him; but it was for the last time on earth, for they met soon thereafter in heaven! And there was Elder G. He was in the prime of life; we sat in the same aisle, met him many a time in the course of years; never spoke to him, never knew his name; but there was holiness and meekness and a high intelligence in his face, which at the first glance or two caused us to put him down in the book of our remembrance as a sainted man. And so it came that, having scattered for the summer and coming back in the autumn, this and that familiar face was seen in the accustomed pew; but the weeks wore on toward winter, and still the gentle, unpretending, unpresuming elder was not there; he had gone to heaven! Just before us there used to come an old lady, only of a Sabbath morning; so decrepit, so feeble, that each day we thought it would be her last in the earthly sanctuary; but she came on. Winter and spring and summer and autumn came, and she did too! as if years ceased to make any further impression on the frail and tottering frame. But we never saw her again.

Not a month ago a mother in Israel sat behind us; no summer's sun, no winter's snow ever kept her away. The petted child of fashion and fortune from earliest infancy, she still knew no deeper joy, and considered it a duty and a privilege, as it was her delight, to mingle her songs and prayers with the Church on earth and in heaven, as a token of her being one of the children of the Great King. Who shall say that she has not met with us for the last time? And there, too, are the refiner brothers. As for many years agone, they walk side by side to the Sabbath sanctuary with the same quick step, the same open, manly, fearless look; their faces always mantled with a smile, as of peace within. Every Sabbath unfailingly have they made their way to the elder's splendid mansion on "the avenue," apparently as indivisible in their home affections as in their business and their princely charities, even to scores of thousands at a time, and that too for these many years past. But what a void there will be when one of the great and noble-hearted twain shall come to the church alone; the "one" brother "taken, the other left," to be lamented as well as "missed" by a Church which numbers half a million of communicants! And not for long shall he who writes sing the last hymn, bow in the last benediction, and turn his back upon the earthly altar to come in again no more forever; for like those before, we too are passing away—

"One by one."

